# Supplementary material for: NextSV: a meta-caller for structural variants from low-coverage long-read sequencing data
Source: BMC Bioinformatics. 2018 May 23;19:180. doi: 10.1186/s12859-018-2207-1 (PMC5966861; doi:10.1186/s12859-018-2207-1)
Supplement: Supplementary file 1 — Tables S1-S24. Performances of BLASR/PBHoney-Spots, BLASR/PBHoney-Tails, BWA/Sniffles, NGMLR/Sniffles and NextSV on the NA12878 genome and the HX1 genome. (PDF 472 kb) [file 12859_2018_2207_MOESM1_ESM.pdf]

Table S1. Performance of BLASR/PBHoney-Spots on NA12878 genome (Deletion calls)

Coverage	Replicate	No. of calls	No. of true positives	Recall	Precision	F1 score
2X	1	913	580	0.277	0.635	0.386
2X	2	874	538	0.257	0.616	0.363
2X	3	869	554	0.265	0.638	0.374
2X	4	898	569	0.272	0.634	0.380
2X	5	906	565	0.270	0.624	0.377
4X	1	1816	1077	0.514	0.593	0.551
4X	2	1805	1073	0.512	0.594	0.550
4X	3	1857	1088	0.520	0.586	0.551
4X	4	1800	1096	0.523	0.609	0.563
4X	5	1780	1080	0.516	0.607	0.558
6X	1	2352	1346	0.643	0.572	0.605
6X	2	2368	1329	0.635	0.561	0.596
6X	3	2427	1372	0.655	0.565	0.607
6X	4	2386	1377	0.658	0.577	0.615
6X	5	2403	1361	0.650	0.566	0.605
8X	1	2716	1493	0.713	0.550	0.621
8X	2	2704	1503	0.718	0.556	0.627
8X	3	2779	1512	0.722	0.544	0.621
8X	4	2729	1520	0.726	0.557	0.630
8X	5	2766	1529	0.730	0.553	0.629
10X	1	2965	1590	0.759	0.536	0.629
10X	2	2941	1589	0.759	0.540	0.631
10X	3	2999	1590	0.759	0.530	0.624
10X	4	2943	1602	0.765	0.544	0.636
10X	5	2995	1603	0.766	0.535	0.630
12X	1	3144	1655	0.790	0.526	0.632
12X	2	3099	1642	0.784	0.530	0.632
12X	3	3136	1636	0.781	0.522	0.626
12X	4	3096	1643	0.785	0.531	0.633
12X	5	3134	1651	0.788	0.527	0.632
15X	1	3257	1697	0.810	0.521	0.634
15X	2	3235	1690	0.807	0.522	0.634
15X	3	3267	1694	0.809	0.519	0.632
15X	4	3230	1686	0.805	0.522	0.633
15X	5	3247	1690	0.807	0.520	0.633

Table S2. Performance of BLASR/PBHoney-Spots on NA12878 genome (Insertion calls)

Coverage	Replicate	No. of calls	No. of true positives	Recall	Precision	F1 score
2X	1	1553	315	0.283	0.203	0.236
2X	2	1517	301	0.270	0.198	0.229
2X	3	1557	317	0.285	0.204	0.237
2X	4	1542	308	0.276	0.200	0.232
2X	5	1565	302	0.271	0.193	0.225
4X	1	3638	611	0.548	0.168	0.257
4X	2	3675	628	0.564	0.171	0.262
4X	3	3555	628	0.564	0.177	0.269
4X	4	3648	641	0.575	0.176	0.269
4X	5	3630	643	0.577	0.177	0.271
6X	1	5246	787	0.706	0.150	0.247
6X	2	5224	789	0.708	0.151	0.249
6X	3	5092	808	0.725	0.159	0.260
6X	4	5150	788	0.707	0.153	0.252
6X	5	5114	785	0.705	0.154	0.252
8X	1	6148	868	0.779	0.141	0.239
8X	2	6163	874	0.785	0.142	0.240
8X	3	6053	886	0.795	0.146	0.247
8X	4	6090	857	0.769	0.141	0.238
8X	5	6071	865	0.776	0.142	0.241
10X	1	6464	907	0.814	0.140	0.239
10X	2	6509	906	0.813	0.139	0.238
10X	3	6426	922	0.828	0.143	0.245
10X	4	6492	896	0.804	0.138	0.236
10X	5	6496	910	0.817	0.140	0.239
12X	1	6609	924	0.829	0.140	0.239
12X	2	6637	926	0.831	0.140	0.239
12X	3	6634	939	0.843	0.142	0.242
12X	4	6583	918	0.824	0.139	0.239
12X	5	6617	926	0.831	0.140	0.240
15X	1	6773	935	0.839	0.138	0.237
15X	2	6808	932	0.837	0.137	0.235
15X	3	6761	945	0.848	0.140	0.240
15X	4	6758	933	0.838	0.138	0.237
15X	5	6744	942	0.846	0.140	0.240

Table S3. Performance of BLASR/PBHoney-Tails on NA12878 genome (Deletion calls)

Coverage	Replicate	No. of calls	No. of true positives	Recall	Precision	F1 score
2X	1	170	102	0.049	0.600	0.090
2X	2	168	104	0.050	0.619	0.092
2X	3	192	104	0.050	0.542	0.091
2X	4	170	95	0.045	0.559	0.084
2X	5	189	116	0.055	0.614	0.102
4X	1	439	246	0.117	0.560	0.194
4X	2	426	234	0.112	0.549	0.186
4X	3	463	251	0.120	0.542	0.196
4X	4	455	258	0.123	0.567	0.202
4X	5	421	247	0.118	0.587	0.196
6X	1	665	355	0.170	0.534	0.257
6X	2	685	356	0.170	0.520	0.256
6X	3	717	382	0.182	0.533	0.272
6X	4	693	364	0.174	0.525	0.261
6X	5	547	279	0.133	0.510	0.211
8X	1	935	464	0.222	0.496	0.306
8X	2	921	446	0.213	0.484	0.296
8X	3	985	505	0.241	0.513	0.328
8X	4	934	460	0.220	0.493	0.304
8X	5	927	461	0.220	0.497	0.305
10X	1	1163	542	0.259	0.466	0.333
10X	2	1161	541	0.258	0.466	0.332
10X	3	1215	577	0.276	0.475	0.349
10X	4	1158	546	0.261	0.472	0.336
10X	5	1174	552	0.264	0.470	0.338
12X	1	1408	613	0.293	0.435	0.350
12X	2	1420	627	0.299	0.442	0.357
12X	3	1443	641	0.306	0.444	0.362
12X	4	918	383	0.183	0.417	0.254
12X	5	1387	612	0.292	0.441	0.352
15X	1	822	282	0.135	0.343	0.193
15X	2	689	222	0.106	0.322	0.160
15X	3	1751	713	0.340	0.407	0.371
15X	4	1799	723	0.345	0.402	0.371
15X	5	1038	379	0.181	0.365	0.242

Table S4. Performance of BLASR/PBHoney-Tails on NA12878 genome (Insertion calls)

Coverage	Replicate	No. of calls	No. of true positives	Recall	Precision	F1 score
2X	1	16	0	0.000	0.000	0.000
2X	2	22	0	0.000	0.000	0.000
2X	3	14	0	0.000	0.000	0.000
2X	4	18	0	0.000	0.000	0.000
2X	5	12	0	0.000	0.000	0.000
4X	1	37	0	0.000	0.000	0.000
4X	2	40	0	0.000	0.000	0.000
4X	3	37	0	0.000	0.000	0.000
4X	4	42	2	0.002	0.048	0.003
4X	5	33	0	0.000	0.000	0.000
6X	1	72	1	0.001	0.014	0.002
6X	2	75	0	0.000	0.000	0.000
6X	3	73	0	0.000	0.000	0.000
6X	4	76	2	0.002	0.026	0.003
6X	5	44	0	0.000	0.000	0.000
8X	1	119	2	0.002	0.017	0.003
8X	2	111	0	0.000	0.000	0.000
8X	3	102	1	0.001	0.010	0.002
8X	4	112	2	0.002	0.018	0.003
8X	5	94	1	0.001	0.011	0.002
10X	1	159	3	0.003	0.019	0.005
10X	2	146	1	0.001	0.007	0.002
10X	3	144	1	0.001	0.007	0.002
10X	4	147	2	0.002	0.014	0.003
10X	5	134	1	0.001	0.007	0.002
12X	1	188	3	0.003	0.016	0.005
12X	2	177	2	0.002	0.011	0.003
12X	3	178	1	0.001	0.006	0.002
12X	4	129	2	0.002	0.016	0.003
12X	5	174	2	0.002	0.011	0.003
15X	1	132	1	0.001	0.008	0.002
15X	2	101	0	0.000	0.000	0.000
15X	3	224	2	0.002	0.009	0.003
15X	4	230	2	0.002	0.009	0.003
15X	5	168	1	0.001	0.006	0.002

Table S5. Performance of BWA/Sniffles on NA12878 genome (Deletion calls)

Coverage	Replicate	No. of calls	No. of true positives	Recall	Precision	F1 score
2X	1	558	462	0.221	0.828	0.348
2X	2	550	462	0.221	0.840	0.349
2X	3	551	454	0.217	0.824	0.343
2X	4	561	467	0.223	0.832	0.352
2X	5	560	468	0.223	0.836	0.353
4X	1	1188	970	0.463	0.816	0.591
4X	2	1167	952	0.455	0.816	0.584
4X	3	1214	979	0.468	0.806	0.592
4X	4	1195	978	0.467	0.818	0.595
4X	5	1204	976	0.466	0.811	0.592
6X	1	1546	1225	0.585	0.792	0.673
6X	2	1587	1247	0.596	0.786	0.678
6X	3	1578	1241	0.593	0.786	0.676
6X	4	1553	1229	0.587	0.791	0.674
6X	5	1595	1253	0.598	0.786	0.679
8X	1	1814	1392	0.665	0.767	0.712
8X	2	1843	1428	0.682	0.775	0.725
8X	3	1835	1416	0.676	0.772	0.721
8X	4	1828	1405	0.671	0.769	0.716
8X	5	1856	1421	0.679	0.766	0.719
10X	1	2004	1498	0.715	0.748	0.731
10X	2	2035	1528	0.730	0.751	0.740
10X	3	2024	1518	0.725	0.750	0.737
10X	4	2040	1527	0.729	0.749	0.739
10X	5	2028	1526	0.729	0.752	0.740
12X	1	2167	1574	0.752	0.726	0.739
12X	2	2167	1590	0.759	0.734	0.746
12X	3	2173	1600	0.764	0.736	0.750
12X	4	2160	1585	0.757	0.734	0.745
12X	5	2165	1595	0.762	0.737	0.749
15X	1	2364	1664	0.795	0.704	0.747
15X	2	2355	1673	0.799	0.710	0.752
15X	3	2338	1655	0.790	0.708	0.747
15X	4	2360	1677	0.801	0.711	0.753
15X	5	2350	1658	0.792	0.706	0.746

Table S6. Performance of BWA/Sniffles on NA12878 genome (Insertion calls)

Coverage	Replicate	No. of calls	No. of true positives	Recall	Precision	F1 score
2X	1	784	106	0.095	0.135	0.112
2X	2	792	100	0.090	0.126	0.105
2X	3	840	105	0.094	0.125	0.107
2X	4	793	93	0.083	0.117	0.098
2X	5	761	96	0.086	0.126	0.102
4X	1	2944	274	0.246	0.093	0.135
4X	2	2891	261	0.234	0.090	0.130
4X	3	2879	246	0.221	0.085	0.123
4X	4	2941	265	0.238	0.090	0.131
4X	5	3013	263	0.236	0.087	0.127
6X	1	5996	400	0.359	0.067	0.113
6X	2	6077	381	0.342	0.063	0.106
6X	3	5998	381	0.342	0.064	0.107
6X	4	6127	390	0.350	0.064	0.108
6X	5	6201	394	0.354	0.064	0.108
8X	1	10201	493	0.443	0.048	0.087
8X	2	10180	495	0.444	0.049	0.088
8X	3	10136	477	0.428	0.047	0.085
8X	4	10298	491	0.441	0.048	0.086
8X	5	10432	495	0.444	0.047	0.086
10X	1	15330	567	0.509	0.037	0.069
10X	2	15309	575	0.516	0.038	0.070
10X	3	15282	563	0.505	0.037	0.069
10X	4	15543	564	0.506	0.036	0.068
10X	5	15516	561	0.504	0.036	0.067
12X	1	21427	620	0.557	0.029	0.055
12X	2	21317	629	0.565	0.030	0.056
12X	3	21400	618	0.555	0.029	0.055
12X	4	21439	633	0.568	0.030	0.056
12X	5	21396	625	0.561	0.029	0.056
15X	1	32350	697	0.626	0.022	0.042
15X	2	32241	686	0.616	0.021	0.041
15X	3	32131	693	0.622	0.022	0.042
15X	4	32267	697	0.626	0.022	0.042
15X	5	32247	693	0.622	0.021	0.042

Table S7. Performance of NGMLR/Sniffles on NA12878 genome (Deletion calls)

Coverage	Replicate	No. of calls	No. of true positives	Recall	Precision	F1 score
2X	1	1039	692	0.330	0.666	0.442
2X	2	953	640	0.306	0.672	0.420
2X	3	1011	686	0.328	0.679	0.442
2X	4	995	672	0.321	0.675	0.435
2X	5	1013	689	0.329	0.680	0.444
4X	1	2064	1324	0.632	0.641	0.637
4X	2	2010	1308	0.625	0.651	0.637
4X	3	2018	1295	0.618	0.642	0.630
4X	4	2036	1315	0.628	0.646	0.637
4X	5	2048	1304	0.623	0.637	0.630
6X	1	2637	1624	0.776	0.616	0.687
6X	2	2603	1615	0.771	0.620	0.688
6X	3	2673	1638	0.782	0.613	0.687
6X	4	2646	1624	0.776	0.614	0.685
6X	5	2675	1638	0.782	0.612	0.687
8X	1	3080	1800	0.860	0.584	0.696
8X	2	3025	1813	0.866	0.599	0.708
8X	3	3080	1830	0.874	0.594	0.707
8X	4	3080	1821	0.870	0.591	0.704
8X	5	3085	1825	0.872	0.592	0.705
10X	1	3344	1894	0.904	0.566	0.697
10X	2	3327	1909	0.912	0.574	0.704
10X	3	3358	1904	0.909	0.567	0.698
10X	4	3368	1922	0.918	0.571	0.704
10X	5	3333	1907	0.911	0.572	0.703
12X	1	3552	1953	0.933	0.550	0.692
12X	2	3562	1960	0.936	0.550	0.693
12X	3	3553	1957	0.935	0.551	0.693
12X	4	3583	1973	0.942	0.551	0.695
12X	5	3573	1965	0.938	0.550	0.693
15X	1	3814	2013	0.961	0.528	0.681
15X	2	3819	2011	0.960	0.527	0.680
15X	3	3829	2004	0.957	0.523	0.677
15X	4	3829	2012	0.961	0.525	0.679
15X	5	3836	2007	0.958	0.523	0.677

Table S8. Performance of NGMLR/Sniffles on NA12878 genome (Insertion calls)

Coverage	Replicate	No. of calls	No. of true positives	Recall	Precision	F1 score
2X	1	1236	265	0.238	0.214	0.226
2X	2	1126	245	0.220	0.218	0.219
2X	3	1231	273	0.245	0.222	0.233
2X	4	1200	258	0.232	0.215	0.223
2X	5	1187	271	0.243	0.228	0.236
4X	1	3142	561	0.504	0.179	0.264
4X	2	3145	573	0.514	0.182	0.269
4X	3	3117	565	0.507	0.181	0.267
4X	4	3140	590	0.530	0.188	0.277
4X	5	3071	580	0.521	0.189	0.277
6X	1	5076	724	0.650	0.143	0.234
6X	2	5094	720	0.646	0.141	0.232
6X	3	5060	726	0.652	0.143	0.235
6X	4	5049	722	0.648	0.143	0.234
6X	5	4921	726	0.652	0.148	0.241
8X	1	7113	800	0.718	0.112	0.194
8X	2	7124	819	0.735	0.115	0.199
8X	3	7118	802	0.720	0.113	0.195
8X	4	7026	780	0.700	0.111	0.192
8X	5	7001	809	0.726	0.116	0.199
10X	1	9278	848	0.761	0.091	0.163
10X	2	9340	844	0.758	0.090	0.161
10X	3	9254	865	0.776	0.093	0.167
10X	4	9131	837	0.751	0.092	0.163
10X	5	9229	860	0.772	0.093	0.166
12X	1	11616	878	0.788	0.076	0.138
12X	2	11719	867	0.778	0.074	0.135
12X	3	11692	902	0.810	0.077	0.141
12X	4	11520	866	0.777	0.075	0.137
12X	5	11583	882	0.792	0.076	0.139
15X	1	15515	909	0.816	0.059	0.109
15X	2	15433	891	0.800	0.058	0.108
15X	3	15366	923	0.829	0.060	0.112
15X	4	15331	912	0.819	0.059	0.111
15X	5	15399	915	0.821	0.059	0.111

Table S9. Performance of NextSV sensitive call set on NA12878 genome (Deletion calls)

Coverage	Replicate	No. of calls	No. of true positives	Recall	Precision	F1 score
2X	1	1434	820	0.392	0.572	0.465
2X	2	1342	762	0.364	0.568	0.444
2X	3	1373	793	0.379	0.578	0.457
2X	4	1363	790	0.377	0.580	0.457
2X	5	1408	818	0.391	0.581	0.467
4X	1	2756	1454	0.694	0.528	0.600
4X	2	2713	1444	0.690	0.532	0.601
4X	3	2777	1447	0.691	0.521	0.594
4X	4	2743	1468	0.701	0.535	0.607
4X	5	2685	1421	0.679	0.529	0.595
6X	1	3550	1741	0.831	0.490	0.617
6X	2	3564	1731	0.827	0.486	0.612
6X	3	3643	1782	0.851	0.489	0.621
6X	4	3590	1765	0.843	0.492	0.621
6X	5	3546	1738	0.830	0.490	0.616
8X	1	4195	1892	0.904	0.451	0.602
8X	2	4128	1908	0.911	0.462	0.613
8X	3	4257	1933	0.923	0.454	0.609
8X	4	4190	1923	0.918	0.459	0.612
8X	5	4175	1907	0.911	0.457	0.608
10X	1	4640	1975	0.943	0.426	0.587
10X	2	4596	1988	0.949	0.433	0.594
10X	3	4689	1977	0.944	0.422	0.583
10X	4	4622	1998	0.954	0.432	0.595
10X	5	4626	1972	0.942	0.426	0.587
12X	1	5034	2022	0.966	0.402	0.567
12X	2	4998	2029	0.969	0.406	0.572
12X	3	5048	2021	0.965	0.400	0.566
12X	4	4876	2030	0.969	0.416	0.582
12X	5	5043	2026	0.968	0.402	0.568
15X	1	5198	2057	0.982	0.396	0.564
15X	2	5117	2053	0.980	0.401	0.569
15X	3	5549	2064	0.986	0.372	0.540
15X	4	5547	2070	0.989	0.373	0.542
15X	5	5277	2056	0.982	0.390	0.558

Table S10. Performance of NextSV sensitive call set on NA12878 genome (Insertion calls)

Coverage	Replicate	No. of calls	No. of true positives	Recall	Precision	F1 score
2X	1	2140	333	0.299	0.156	0.205
2X	2	2043	321	0.288	0.157	0.203
2X	3	2125	346	0.311	0.163	0.214
2X	4	2099	329	0.295	0.157	0.205
2X	5	2107	333	0.299	0.158	0.207
4X	1	5247	657	0.590	0.125	0.207
4X	2	5283	677	0.608	0.128	0.212
4X	3	5172	666	0.598	0.129	0.212
4X	4	5244	686	0.616	0.131	0.216
4X	5	5148	683	0.613	0.133	0.218
6X	1	8203	836	0.750	0.102	0.179
6X	2	8193	837	0.751	0.102	0.180
6X	3	8076	848	0.761	0.105	0.185
6X	4	8108	839	0.753	0.103	0.182
6X	5	7934	836	0.750	0.105	0.185
8X	1	10821	926	0.831	0.086	0.155
8X	2	10830	937	0.841	0.087	0.157
8X	3	10759	932	0.837	0.087	0.157
8X	4	10735	918	0.824	0.086	0.155
8X	5	10646	921	0.827	0.087	0.157
10X	1	13131	973	0.873	0.074	0.137
10X	2	13226	977	0.877	0.074	0.136
10X	3	13061	987	0.886	0.076	0.139
10X	4	13096	975	0.875	0.074	0.137
10X	5	13100	976	0.876	0.075	0.137
12X	1	15501	1003	0.900	0.065	0.121
12X	2	15634	1012	0.908	0.065	0.121
12X	3	15580	1015	0.911	0.065	0.122
12X	4	15433	1000	0.898	0.065	0.121
12X	5	15467	1000	0.898	0.065	0.121
15X	1	19396	1023	0.918	0.053	0.100
15X	2	19385	1023	0.918	0.053	0.100
15X	3	19311	1034	0.928	0.054	0.101
15X	4	19271	1025	0.920	0.053	0.101
15X	5	19306	1026	0.921	0.053	0.100

Table S11. Performance of NextSV stringent call set on NA12878 genome (Deletion calls)

Coverage	Replicate	No. of calls	No. of true positives	Recall	Precision	F1 score
2X	1	670	537	0.256	0.801	0.389
2X	2	640	509	0.243	0.795	0.372
2X	3	686	541	0.258	0.789	0.389
2X	4	692	540	0.258	0.780	0.388
2X	5	687	543	0.259	0.790	0.391
4X	1	1504	1142	0.545	0.759	0.635
4X	2	1455	1116	0.533	0.767	0.629
4X	3	1478	1128	0.539	0.763	0.632
4X	4	1464	1137	0.543	0.777	0.639
4X	5	1491	1142	0.545	0.766	0.637
6X	1	1938	1452	0.693	0.749	0.720
6X	2	1916	1432	0.684	0.747	0.714
6X	3	1991	1468	0.701	0.737	0.719
6X	4	1949	1454	0.694	0.746	0.719
6X	5	1943	1437	0.686	0.740	0.712
8X	1	2253	1645	0.786	0.730	0.757
8X	2	2253	1657	0.791	0.735	0.762
8X	3	2278	1673	0.799	0.734	0.765
8X	4	2273	1662	0.794	0.731	0.761
8X	5	2302	1674	0.799	0.727	0.762
10X	1	2439	1750	0.836	0.718	0.772
10X	2	2452	1768	0.844	0.721	0.778
10X	3	2463	1767	0.844	0.717	0.776
10X	4	2459	1776	0.848	0.722	0.780
10X	5	2459	1759	0.840	0.715	0.773
12X	1	2570	1819	0.869	0.708	0.780
12X	2	2577	1818	0.868	0.705	0.778
12X	3	2572	1823	0.871	0.709	0.781
12X	4	2423	1742	0.832	0.719	0.771
12X	5	2551	1818	0.868	0.713	0.783
15X	1	2428	1746	0.834	0.719	0.772
15X	2	2416	1722	0.822	0.713	0.764
15X	3	2664	1874	0.895	0.703	0.788
15X	4	2671	1876	0.896	0.702	0.787
15X	5	2498	1768	0.844	0.708	0.770

Table S12. Performance of NextSV stringent call set on NA12878 genome (Insertion calls)

Coverage	Replicate	No. of calls	No. of true positives	Recall	Precision	F1 score
2X	1	665	247	0.222	0.371	0.278
2X	2	616	225	0.202	0.365	0.260
2X	3	674	244	0.219	0.362	0.273
2X	4	657	237	0.213	0.361	0.268
2X	5	656	240	0.215	0.366	0.271
4X	1	1558	515	0.462	0.331	0.385
4X	2	1569	524	0.470	0.334	0.391
4X	3	1528	527	0.473	0.345	0.399
4X	4	1571	546	0.490	0.348	0.407
4X	5	1577	540	0.485	0.342	0.401
6X	1	2166	675	0.606	0.312	0.412
6X	2	2172	672	0.603	0.309	0.409
6X	3	2120	686	0.616	0.324	0.424
6X	4	2138	672	0.603	0.314	0.413
6X	5	2130	675	0.606	0.317	0.416
8X	1	2508	743	0.667	0.296	0.410
8X	2	2526	756	0.679	0.299	0.415
8X	3	2473	756	0.679	0.306	0.422
8X	4	2450	721	0.647	0.294	0.405
8X	5	2483	753	0.676	0.303	0.419
10X	1	2698	783	0.703	0.290	0.411
10X	2	2710	774	0.695	0.286	0.405
10X	3	2699	800	0.718	0.296	0.420
10X	4	2614	760	0.682	0.291	0.408
10X	5	2699	794	0.713	0.294	0.416
12X	1	2833	800	0.718	0.282	0.405
12X	2	2824	783	0.703	0.277	0.398
12X	3	2849	825	0.741	0.290	0.416
12X	4	2754	786	0.706	0.285	0.406
12X	5	2829	808	0.725	0.286	0.410
15X	1	2972	822	0.738	0.277	0.402
15X	2	2918	801	0.719	0.275	0.397
15X	3	2953	834	0.749	0.282	0.410
15X	4	2965	822	0.738	0.277	0.403
15X	5	2942	833	0.748	0.283	0.411

Table S13. Performance of BLASR/PBHoney-Spots on HX1 genome (Deletion calls)

Coverage	Replicate	No. of calls	No. of true positives	Recall	Precision	F1 score
6X	1	2956	1669	0.699	0.565	0.625
6X	2	3021	1694	0.710	0.561	0.626
6X	3	2961	1668	0.699	0.563	0.624
6X	4	2990	1672	0.700	0.559	0.622
6X	5	2962	1672	0.700	0.564	0.625
10X	1	3747	1908	0.799	0.509	0.622
10X	2	3733	1893	0.793	0.507	0.619
10X	3	3729	1890	0.792	0.507	0.618
10X	4	3717	1886	0.790	0.507	0.618
10X	5	3781	1890	0.792	0.500	0.613
15X	1	4167	1958	0.820	0.470	0.597
15X	2	4172	1979	0.829	0.474	0.603
15X	3	4164	1970	0.825	0.473	0.601
15X	4	4205	1971	0.826	0.469	0.598
15X	5	4137	1964	0.823	0.475	0.602

Table S14. Performance of BLASR/PBHoney-Spots on HX1 genome (Insertion calls)

Coverage	Replicate	No. of calls	No. of true positives	Recall	Precision	F1 score
6X	1	4877	2091	0.712	0.429	0.535
6X	2	4781	2140	0.729	0.448	0.555
6X	3	4848	2129	0.725	0.439	0.547
6X	4	4753	2106	0.717	0.443	0.548
6X	5	4814	2075	0.707	0.431	0.535
10X	1	5998	2383	0.811	0.397	0.533
10X	2	6028	2405	0.819	0.399	0.537
10X	3	5975	2397	0.816	0.401	0.538
10X	4	6020	2399	0.817	0.399	0.536
10X	5	6003	2386	0.812	0.397	0.534
15X	1	6244	2419	0.824	0.387	0.527
15X	2	6229	2412	0.821	0.387	0.526
15X	3	6217	2415	0.822	0.388	0.528
15X	4	6215	2402	0.818	0.386	0.525
15X	5	6311	2404	0.819	0.381	0.520

Table S15. Performance of BLASR/PBHoney-Tails on HX1 genome (Deletion calls)

Coverage	Replicate	No. of calls	No. of true positives	Recall	Precision	F1 score
6X	1	419	265	0.111	0.632	0.189
6X	2	434	293	0.123	0.675	0.208
6X	3	438	281	0.118	0.642	0.199
6X	4	462	285	0.119	0.617	0.200
6X	5	449	289	0.121	0.644	0.204
10X	1	748	422	0.177	0.564	0.269
10X	2	768	457	0.191	0.595	0.290
10X	3	730	438	0.183	0.600	0.281
10X	4	757	450	0.189	0.594	0.286
10X	5	749	443	0.186	0.591	0.283
15X	1	1126	595	0.249	0.528	0.339
15X	2	1079	595	0.249	0.551	0.343
15X	3	1093	599	0.251	0.548	0.344
15X	4	1050	577	0.242	0.550	0.336
15X	5	1091	598	0.251	0.548	0.344

Table S16. Performance of BLASR/PBHoney-Tails on HX1 genome (Insertion calls)

Coverage	Replicate	No. of calls	No. of true positives	Recall	Precision	F1 score
6X	1	30	6	0.002	0.200	0.004
6X	2	28	5	0.002	0.179	0.003
6X	3	32	7	0.002	0.219	0.005
6X	4	31	6	0.002	0.194	0.004
6X	5	29	4	0.001	0.138	0.003
10X	1	60	16	0.005	0.267	0.011
10X	2	48	13	0.004	0.271	0.009
10X	3	56	9	0.003	0.161	0.006
10X	4	58	13	0.004	0.224	0.009
10X	5	58	15	0.005	0.259	0.010
15X	1	91	20	0.007	0.220	0.013
15X	2	91	23	0.008	0.253	0.015
15X	3	92	25	0.009	0.272	0.017
15X	4	87	26	0.009	0.299	0.017
15X	5	89	24	0.008	0.270	0.016

Table S17. Performance of BWA/Sniffles on HX1 genome (Deletion calls)

Coverage	Replicate	No. of calls	No. of true positives	Recall	Precision	F1 score
6X	1	1668	1295	0.543	0.776	0.639
6X	2	1717	1323	0.554	0.771	0.645
6X	3	1698	1316	0.551	0.775	0.644
6X	4	1725	1328	0.556	0.770	0.646
6X	5	1714	1314	0.550	0.767	0.641
10X	1	2223	1558	0.653	0.701	0.676
10X	2	2233	1575	0.660	0.705	0.682
10X	3	2220	1559	0.653	0.702	0.677
10X	4	2254	1571	0.658	0.697	0.677
10X	5	2228	1563	0.655	0.702	0.677
15X	1	2774	1696	0.711	0.611	0.657
15X	2	2713	1718	0.720	0.633	0.674
15X	3	2702	1680	0.704	0.622	0.660
15X	4	2721	1686	0.706	0.620	0.660
15X	5	2702	1679	0.703	0.621	0.660

Table S18. Performance of BWA/Sniffles on HX1 genome (Insertion calls)

Coverage	Replicate	No. of calls	No. of true positives	Recall	Precision	F1 score
6X	1	4019	400	0.136	0.100	0.115
6X	2	4131	422	0.144	0.102	0.119
6X	3	4073	403	0.137	0.099	0.115
6X	4	4061	423	0.144	0.104	0.121
6X	5	4088	409	0.139	0.100	0.116
10X	1	10228	598	0.204	0.058	0.091
10X	2	10260	626	0.213	0.061	0.095
10X	3	10338	594	0.202	0.057	0.089
10X	4	10170	609	0.207	0.060	0.093
10X	5	10184	605	0.206	0.059	0.092
15X	1	21426	764	0.260	0.036	0.063
15X	2	21532	736	0.251	0.034	0.060
15X	3	21582	775	0.264	0.036	0.063
15X	4	21578	770	0.262	0.036	0.063
15X	5	21428	775	0.264	0.036	0.064

Table S19. Performance of NGMLR/Sniffles on HX1 genome (Deletion calls)

Coverage	Replicate	No. of calls	No. of true positives	Recall	Precision	F1 score
6X	1	2731	1885	0.790	0.690	0.737
6X	2	2785	1927	0.807	0.692	0.745
6X	3	2754	1889	0.791	0.686	0.735
6X	4	2796	1920	0.804	0.687	0.741
6X	5	2767	1907	0.799	0.689	0.740
10X	1	3452	2178	0.912	0.631	0.746
10X	2	3470	2184	0.915	0.629	0.746
10X	3	3470	2185	0.915	0.630	0.746
10X	4	3493	2187	0.916	0.626	0.744
10X	5	3490	2203	0.923	0.631	0.750
15X	1	4009	2293	0.961	0.572	0.717
15X	2	3989	2290	0.959	0.574	0.718
15X	3	3970	2289	0.959	0.577	0.720
15X	4	3973	2286	0.958	0.575	0.719
15X	5	3954	2281	0.956	0.577	0.719

Table S20. Performance of NGMLR/Sniffles on HX1 genome (Insertion calls)

Coverage	Replicate	No. of calls	No. of true positives	Recall	Precision	F1 score
6X	1	4694	1674	0.570	0.357	0.439
6X	2	4724	1666	0.567	0.353	0.435
6X	3	4664	1677	0.571	0.360	0.441
6X	4	4733	1688	0.575	0.357	0.440
6X	5	4752	1670	0.569	0.351	0.434
10X	1	8508	2013	0.685	0.237	0.352
10X	2	8813	2002	0.682	0.227	0.341
10X	3	8593	2025	0.689	0.236	0.351
10X	4	8690	2010	0.684	0.231	0.346
10X	5	8789	2011	0.685	0.229	0.343
15X	1	14434	2167	0.738	0.150	0.249
15X	2	14404	2182	0.743	0.151	0.252
15X	3	14617	2183	0.743	0.149	0.249
15X	4	14665	2181	0.743	0.149	0.248
15X	5	14561	2168	0.738	0.149	0.248

Table S21. Performance of NextSV sensitive call set on HX1 genome (Deletion calls)

Coverage	Replicate	No. of calls	No. of true positives	Recall	Precision	F1 score
6X	1	3967	2029	0.850	0.511	0.639
6X	2	4036	2070	0.867	0.513	0.645
6X	3	3990	2036	0.853	0.510	0.639
6X	4	4051	2056	0.861	0.508	0.639
6X	5	4005	2068	0.866	0.516	0.647
10X	1	5135	2276	0.953	0.443	0.605
10X	2	5134	2274	0.953	0.443	0.605
10X	3	5127	2275	0.953	0.444	0.606
10X	4	5115	2277	0.954	0.445	0.607
10X	5	5193	2294	0.961	0.442	0.605
15X	1	6083	2355	0.987	0.387	0.556
15X	2	6003	2364	0.990	0.394	0.564
15X	3	6004	2362	0.990	0.393	0.563
15X	4	6024	2357	0.987	0.391	0.560
15X	5	6000	2358	0.988	0.393	0.562

Table S22. Performance of NextSV sensitive call set on HX1 genome (Insertion calls)

Coverage	Replicate	No. of calls	No. of true positives	Recall	Precision	F1 score
6X	1	7422	2313	0.788	0.312	0.447
6X	2	7379	2332	0.794	0.316	0.452
6X	3	7388	2327	0.792	0.315	0.451
6X	4	7384	2313	0.788	0.313	0.448
6X	5	7426	2292	0.780	0.309	0.442
10X	1	11831	2654	0.904	0.224	0.359
10X	2	12151	2649	0.902	0.218	0.351
10X	3	11874	2650	0.902	0.223	0.358
10X	4	12046	2654	0.904	0.220	0.354
10X	5	12121	2654	0.904	0.219	0.353
15X	1	17841	2753	0.937	0.154	0.265
15X	2	17760	2769	0.943	0.156	0.268
15X	3	17926	2757	0.939	0.154	0.264
15X	4	17994	2735	0.931	0.152	0.261
15X	5	17995	2748	0.936	0.153	0.263

Table S23. Performance of NextSV stringent call set on HX1 genome (Deletion calls)

Coverage	Replicate	No. of calls	No. of true positives	Recall	Precision	F1 score
6X	1	2005	1640	0.687	0.818	0.747
6X	2	2050	1664	0.697	0.812	0.750
6X	3	2007	1627	0.682	0.811	0.741
6X	4	2039	1646	0.690	0.807	0.744
6X	5	2027	1628	0.682	0.803	0.738
10X	1	2486	1930	0.809	0.776	0.792
10X	2	2477	1916	0.803	0.774	0.788
10X	3	2462	1918	0.804	0.779	0.791
10X	4	2503	1921	0.805	0.767	0.786
10X	5	2504	1924	0.806	0.768	0.787
15X	1	2651	1997	0.837	0.753	0.793
15X	2	2681	2016	0.845	0.752	0.796
15X	3	2681	2022	0.847	0.754	0.798
15X	4	2665	2002	0.839	0.751	0.793
15X	5	2646	2003	0.839	0.757	0.796

Table S24. Performance of NextSV stringent call set on HX1 genome (Insertion calls)

Coverage	Replicate	No. of calls	No. of true positives	Recall	Precision	F1 score
6X	1	2166	1456	0.496	0.672	0.571
6X	2	2144	1477	0.503	0.689	0.581
6X	3	2149	1483	0.505	0.690	0.583
6X	4	2119	1485	0.506	0.701	0.587
6X	5	2159	1456	0.496	0.674	0.571
10X	1	2714	1753	0.597	0.646	0.620
10X	2	2721	1766	0.601	0.649	0.624
10X	3	2733	1777	0.605	0.650	0.627
10X	4	2692	1760	0.599	0.654	0.625
10X	5	2703	1751	0.596	0.648	0.621
15X	1	2899	1846	0.629	0.637	0.633
15X	2	2937	1840	0.626	0.626	0.626
15X	3	2970	1853	0.631	0.624	0.627
15X	4	2943	1858	0.633	0.631	0.632
15X	5	2933	1833	0.624	0.625	0.625
